# Safety of leadless versus transvenous pacemakers in patients with low body surface area: a matched-pair analysis

**DOI:** 10.1186/s12872-026-05526-0

**Published:** 2026-01-16

**Authors:** Sirin Apiyasawat, Natcha Soontornmanokati, Tachapong Ngarmukos, Nilubon Methachittiphan

**Affiliations:** Faculty of Medicine Ramathibodi Hospital, Mahidol University, Bangkok, Thailand

**Keywords:** Leadless pacemaker, Low body surface area, Transvenous pacemaker, Asian population, Safety comparison, Matched-pair analysis, Major complications, Charlson comorbidity index

## Abstract

**Background:**

Leadless pacemakers (LPs) avoid lead- and pocket-related issues but require large venous access, which raises safety concerns in patients with a small body surface area (BSA), particularly in Asian populations. We defined low BSA as < 1.46 m², based on validated 3D-derived anthropometric standards for Chinese adults.

**Objective:**

To assess procedural complication rates of LP implantation in patients with low BSA.

**Methods:**

We analyzed LP implantations from 2016 to 2025 at a single center in Thailand. Of 59 patients, 25 (42.4%) had low BSA. We conducted two comparisons: (1) low- vs. normal/high-BSA LP patients and (2) low-BSA LP patients with a 1:2 age-, sex-, and BSA-matched transvenous pacemaker (TVP) cohort. The primary outcome was major device-related complications.

**Results:**

LP patients were elderly (median 79 years) with high comorbidity (median Charlson Comorbidity Index [CCI], 7.0). In the matched analysis, low-BSA LP patients had a significantly higher comorbidity burden than TVP controls (median CCI, 7.0 vs. 5.0; *P* = 0.002). The 3.8-year cumulative incidence of major complications did not differ between low- vs. normal/high-BSA LP patients (8.0% vs. 8.8%, *P* = 0.39) and between low-BSA LP patients vs. matched TVP controls (8.0% vs. 14.0%, *P* = 0.91). Adjusted analyses revealed no significant association between device type and complications, although the confidence intervals were wide.

**Conclusion:**

In this exploratory analysis, patients with low-BSA showed no increased risk of major complications following LP implantation. However, given the small sample size and pronounced selection bias, these findings should be interpreted with caution.

**Supplementary Information:**

The online version contains supplementary material available at 10.1186/s12872-026-05526-0.

## Background

Leadless pacemakers (LPs) offer several advantages over conventional transvenous pacemakers (TVPs). Without leads or subcutaneous pockets, they mitigate primary complications associated with the transvenous system, including pocket infections and lead integrity issues. LPs also represent the preferred option for patients with limited upper-extremity venous access. In the pre-approval study of Micra LP, the incidence of major complications in the LP group was significantly lower than that of control TVP patients in both the short- and long-term periods [1, 2]. Real-world data [3] further demonstrated a 32% reduction in chronic complications and a 41% reduction in reinterventions with Micra LP compared to TVPs at 3 years. These safety benefits extend to high-risk subgroups [4], such as patients with end-stage renal disease or chronic obstructive pulmonary disease, who experienced lower rates of chronic complications and reinterventions than matched TVP controls.

A notable procedural challenge for LP implantation is the requirement of a large-bore sheath, specifically a 29 Fr or 9 mm outer diameter, for the Micra system. This sheath size is a concern for patients with small body habitus, especially in Asian populations. Difficulties may arise during catheter introduction into the central vein and right ventricle manipulation, leading to complications, including vascular injury or cardiac perforation. For instance, a case of Micra LP implantation in a child weighing 16 kg reported some difficulties with catheter manipulation in the right ventricle and a vascular thrombus as a complication after the procedure [5]. In a national inpatient database of patients who underwent LP implantation in the United States [6], patients with overweight and obesity experienced fewer pericardial complications and bleeding than those with normal weight. Similarly, in a cohort from Hong Kong [7], individuals with low body weight and low body mass index (BMI) demonstrated poorer safety outcomes than those with higher weight or BMI. However, the heterogeneity of these studies—ranging from Western to Asian populations, adults to children, and varying comorbidities—limits their generalizability. Conversely, small body habitus has been consistently identified in large-scale registries as a risk factor for TVP complications. Data from the Danish Pacemaker Registry (*n* = 5,918) [8] showed that underweight patients face a 1.5-fold increased risk of major complications, including pneumothorax and perforation. Similarly, the Mayo Clinic pacemaker database (*n* = 4,280) [9] reported 1.6-fold increased odds of cardiac perforation in patients with a BMI < 20 kg/m^2^. Given these competing risks, it remains unclear whether LPs or TVPs offer a superior safety profile for patients with small body habitus.

To address this, an accurate anthropometric metric is essential. While BMI is the most commonly used measure, body surface area (BSA) may serve as a superior metric for evaluating procedural risk in this context. BSA correlates more strongly than BMI or weight alone with the diameter of the common femoral vein [10, 11]—the critical access point for LP delivery—and with cardiac chamber dimensions [12], which dictate the maneuvering space within the right ventricle. Therefore, BSA may provide a more physiologically relevant index for predicting vascular and intracardiac procedural difficulty.

In this study, we investigated the procedural safety and clinical outcomes of LP implantation in patients with low BSA. We performed two comparative analyses: (1) an internal comparison (within the LP cohort) assessing outcomes between patients with low BSA and those with normal or high BSA, and (2) an external comparison assessing outcomes between the low-BSA LP cohort and a 1:2 age-, sex-, and BSA-matched control group of patients receiving TVPs. We hypothesized that LPs might serve as a feasible alternative in this population; however, given the paucity of existing data and the limited sample size, this study was designed as an exploratory analysis to characterize safety signals rather than to definitively establish clinical equivalence.

## Methods

### Study design

**Fig. 1:**
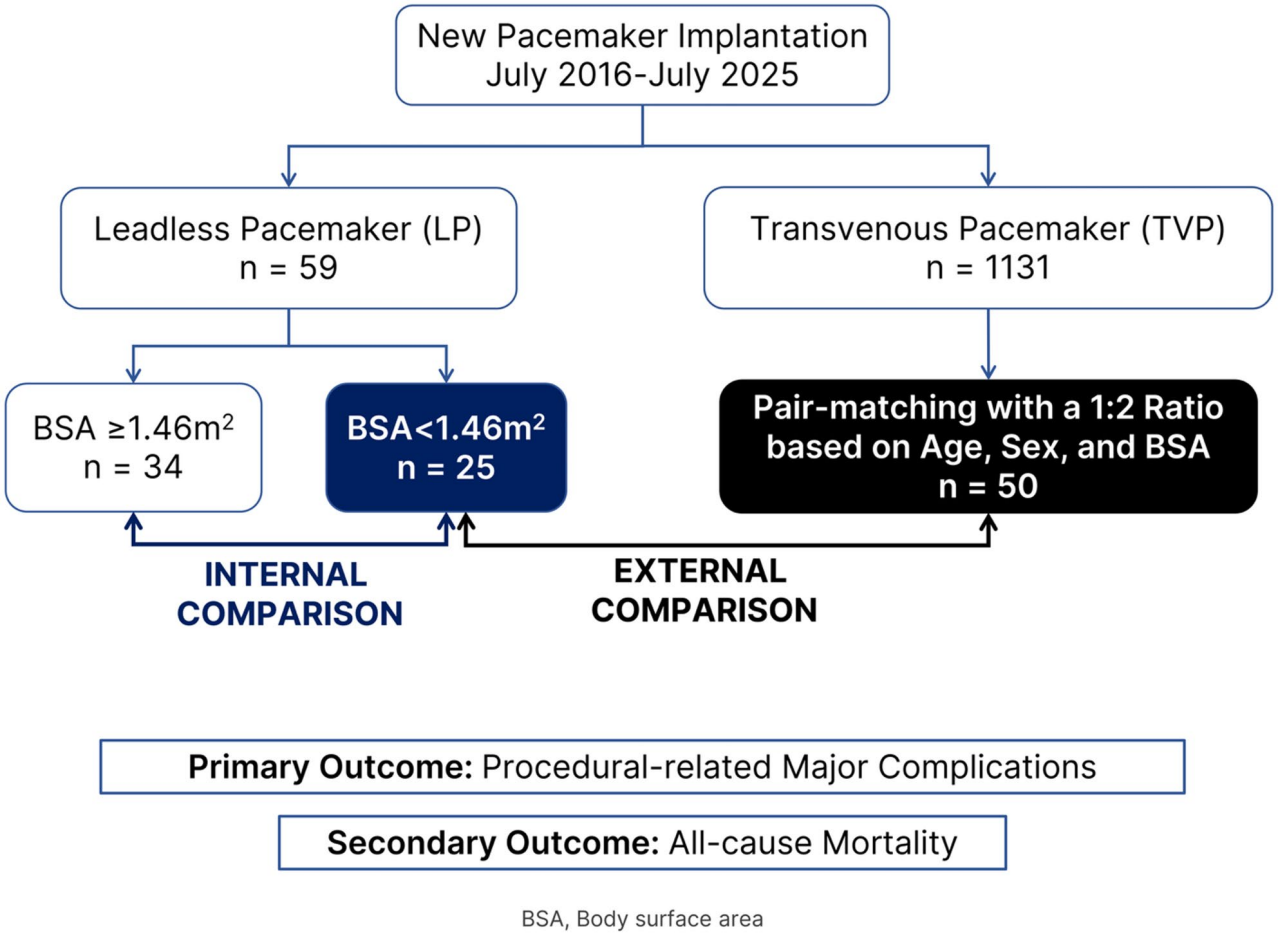
Study flow for a trial of safety of leadless vs. transvenous pacemakers in patients with low body surface area: a matched-pair analysis. Abbreviations: BSA, body surface area; LP, leadless pacemaker; TVP, transvenous pacemaker

This retrospective cohort study was conducted at a single tertiary center in Thailand. All LP implantations from the first case in July 2016 to July 2025 were reviewed. All new TVP implantations performed during the same period were identified as the matched control. This study involved two primary analyses (Fig. 1).

1. An internal comparison within the LP cohort assessing outcomes between patients with low BSA (low-BSA LP) and those with normal or high BSA (normal/high-BSA LP).
2. An external comparison assessing outcomes between the low-BSA LP cohort and a 1:2 age-, sex-, and BSA-matched control group of patients receiving TVPs.

This study was approved by the Institutional Review Board of the Department of Medicine, Faculty of Medicine, Ramathibodi Hospital, Mahidol University (COA No. MURA2025/700). Data were extracted from the electronic medical records and de-identified before the analysis. The requirement for informed consent was waived.

### Patient population

Patients aged ≥ 18 years who underwent a new LP and TVP implantation between July 2016 and July 2025 were identified. All the patients who underwent LP were included in the internal analysis. The patients were categorized into two groups: those with a low BSA (< 1.46 m²) and those with a normal/high BSA (≥ 1.46 m²) (see definition and calculation method below).

For external comparison, each patient in the low-BSA LP group was pair-matched with two control patients from the TVP cohort (1:2 ratio). Matching was performed sequentially using a greedy algorithm without replacement to maximize control pool utilization while ensuring the independence of selected controls. The matching criteria were applied in the following order of priority: (1) BSA (caliper matching), (2) sex (exact match), and (3) age (caliper matching). The caliper width of 0.25 standard deviations, derived from the low-BSA LP group, was applied to each matching variable (BSA and age). Given that body habitus was the primary variable of interest, priority was given to eligible TVP controls with the BSA closest to the corresponding LP case.

### BSA calculation and definition of low BSA

BSA was estimated using the three-dimensionally derived formula developed by Yu et al. [13]:

$$\mathrm{BSA}=0.015925\;\times\;\left(\mathrm{Height}\left(\mathrm{cm}\right)\;\times\;\mathrm{Weight}\;\left(\mathrm{kg}\right)\right)^{1/2}$$

 

This formula offers greater accuracy than traditional methods. A cut-off value of 1.46 m² was selected to define low BSA. This cutoff was based on data from a study involving Chinese participants aged 20–91.^13^ Here, BSA was calculated using the above-mentioned formula, and the value of 1.46 m² represented the median BSA of the lowest-mass subgroup (females aged 72–91 years). Given the shared Asian anthropometric phenotype, this value serves as a relevant reference for our population. Furthermore, this cutoff is consistent with local normative data; a study of 60 healthy Thai volunteers reported a mean BSA of 1.63 ± 0.17 m² [14]. Notably, our chosen cutoff of 1.46 m² aligns exactly with one standard deviation below the mean in this Thai cohort. Although the Thai dataset is small and the calculation was made using a traditional formula, the alignment with the larger Chinese cohort reinforces the validity of this threshold as a definition of small body habitus in our study.

The Charlson Comorbidity Index (CCI) was calculated for each patient to quantify baseline comorbidities. This is a widely recognized scoring system for classifying prognostic comorbidities. The score is based on 19 different comorbidities [15], with a CCI score of 5 or higher, indicating a severe level of comorbidities associated with an increased mortality risk.

Significant tricuspid valve disease was defined as moderate or greater valvular regurgitation or stenosis, or a history of tricuspid valve surgery or intervention.

### Procedures

All LPs were Micra Devices (Medtronic Inc.). These models included MC1VR01, MC1AVR1, MC2VR01, and MC2AVR1. Implantation location was determined using post-implantation chest radiography. The interventricular septum was divided into two equal halves: devices in the lower half were classified as the apex/apical septum, and those in the upper half as the mid/high septum. This method has been previously validated against cardiac computed tomography, demonstrating a 97% concordance rate and a high interobserver correlation coefficient of 0.86 for distinguishing between apical and non-apical septal positions [16]. 

Single- or dual-chamber pacemakers from all major manufacturers (Abbott, Biotronic, Boston Scientific, and Medtronic) were used. All procedures were performed by 3 experienced electrophysiologists (NM, SA, and TN).

### Follow-up and outcomes

At our institution, patients are routinely followed up for wound care, device function, and adverse events at 1 week, 4 weeks, and at least every 6 months after implantation. All follow-up reports were obtained from the electronic medical records.

The primary outcome was the incidence of major device-related complications, as defined by the criteria used in the Micra Transcatheter Pacing Study [1]. A major complication was defined as system- or procedure-related adverse events resulting in death, permanent device function loss, hospitalization, prolonged hospitalization (≥ 48 h), or system revision. These included cardiac complications, defined as cardiac perforation, pericardial effusion, or cardiac tamponade; and vascular complications, defined as puncture site events (groin hematoma, pseudoaneurysm, or arteriovenous fistula) that required surgical repair, percutaneous intervention, or blood transfusion.

Complications were classified as acute if they occurred within 4 weeks of the procedure and late if they occurred after 4 weeks. The secondary outcome was all-cause mortality. Clinical outcomes were adjudicated through a comprehensive manual review of electronic medical records by the study authors.

To ensure data completeness for acute complications, loss to follow-up prior to 4 weeks was a pre-specified exclusion criterion, except for mortality events. The 4-week threshold was selected to align with our institution’s routine follow-up schedule and to capture the incidence of acute complications within this timeframe.

### Statistical analysis

All statistical analyses were performed using Python (version 3.11.10). Survival analyses and statistical tests were conducted using the lifelines (version 0.30.0) and SciPy (version 1.14.1) libraries, respectively. Statistical significance was defined as a two-sided *P* value of < 0.05. Given the exploratory nature of this trial, no adjustments were made for multiple comparisons.

The baseline characteristics and patient outcomes were summarized using descriptive statistics. Continuous variables were assessed for normality using visual inspection and the Shapiro-Wilk test. As all continuous variables were found to be non-normally distributed, data are expressed as medians along with their interquartile ranges, reported as Q1–Q3. Categorical variables are presented as frequencies and percentages.

For the internal comparison of low- and normal/high-BSA, the Mann–Whitney U test was used to compare continuous variables. Categorical variables were compared using Pearson’s chi-square test; however, Fisher’s exact test was employed whenever expected cell counts were fewer than five. For the outcome analysis, a competing risks framework was used to account for death as a competing risk factor for major complications and major complications as a competing risk factor for death. Cumulative incidence was calculated using the Aalen–Johansen (AJ) estimator. Differences in the cumulative incidence of events between the low- and normal/high-BSA groups were compared using Gray’s test.

For the external comparison of the low-BSA LP cohort and a 1:2 age-, sex-, and BSA-matched TVP cohort, baseline characteristics were compared using conditional logistic regression, stratified by a matched set identifier. In this analysis, regression was conditioned on a unique matched-pair identifier. For the outcome analysis, the cumulative incidence was calculated using the AJ estimator to account for death as a competing risk factor for major complications and major complications as a competing risk factor for death. To evaluate the association between pacemaker type and outcome in the matched cohort analysis, we employed a stratified cause-specific Cox proportional hazards model. This analysis was stratified by a matched set identifier to account for the study design and was analyzed in a cause-specific manner to account for death as a competing risk factor for major complications and major complications as a competing risk factor for death. This cause-specific approach was selected to isolate the etiological association between device type and complications, independent of the competing risk of mortality. Additionally, a multivariable model was developed to adjust for the CCI, which was significantly different between the groups at baseline. To prevent overadjustment, CCI was the only variable included in the multivariable model. Variables used in the matching algorithm (age, sex, and BSA) were explicitly excluded. The proportional hazards assumption was verified for all covariates using Schoenfeld residuals, and no violations were identified. The results are presented as hazard ratios (HRs) and adjusted hazard ratios (aHRs) with 95% confidence intervals (CIs).

## Results

### Baseline characteristics of all patients

Overall, 59 patients with newly implanted LPs were identified. No patients were excluded due to loss of follow-up within 4 weeks after implantation, as all patients had data beyond this duration or reached a mortality endpoint. The baseline patient characteristics are presented in Table 1.

**Table 1:** Baseline characteristics of all patients with leadless pacemakers (*N* = 59)

Characteristic	Data Value
Age, median (IQR), year	79.0 (69.0–83.0)
Women, No. (%)	31 (52.5)
Weight, median (IQR), kg	55.5 (45.0–62.9)
Height, median (IQR), m	1.58 (1.52–1.65)
Body surface area, median (IQR), m^2^	1.48 (1.35–1.62)
Body mass index, median (IQR), kg/m^2^	21.62 (19.62–23.72)
Charlson Comorbidity Index, median (IQR)	7.0 (5.5–8.0)
Atrial fibrillation, No. (%)	27 (45.8)
Chronic kidney disease, No. (%)	
Stage 1	3 (5.1)
Stage 2	18 (30.5)
Stage 3	3 (5.1)
Stage 4	2 (3.4)
Stage 5	33 (55.9)
Coronary artery disease, No. (%)	20 (33.9)
Congestive heart failure, No. (%)	19 (32.2)
Cerebrovascular accident, No. (%)	10 (16.9)
Chronic obstructive pulmonary disease, No. (%)	1 (1.7)
Chronic liver disease, No. (%)	3 (5.1)
Connective tissue disease, No. (%)	2 (3.4)
Dementia, No. (%)	8 (13.5)
Diabetes, No. (%)	27 (45.8)
Hypertension, No. (%)	17 (28.8)
Lymphoma or leukemia, No. (%)	1 (1.7)
Malignancy, No. (%)	
Without metastasis	5 (8.5)
With metastasis	1 (1.7)
Peripheral arterial disease, No. (%)	8 (13.5)
Peptic ulcer, No. (%)	14 (23.7)
Significant tricuspid valve disease, No. (%)	
Moderate to severe regurgitation	11 (18.6)
Surgical repair	1 (1.7)
Use of antiplatelet, No. (%)	
Single agent	17 (28.8)
Dual agents	8 (13.5)
Use of oral anticoagulant, No. (%)	
Direct oral anticoagulant	8 (13.5)
Vitamin K antagonist	18 (30.5)
Indication for pacemaker, No. (%)	
Sinus node dysfunction	23 (39.0)
High-grade atrioventricular block	35 (59.3)
Both	1 (1.7)
Specific indication for leadless pacemaker, No. (%)	
Limited access	35 (59.3)
High infection risk	8 (13.5)
Post-tricuspid valve intervention	2 (3.4)
Implantation location, No. (%)	
Apical/apical septum	32 (54.2)
Mid/high septum	27 (45.8)

*Abbreviations*: *IQR* interquartile range

The median age was 79 (69–83) years, and the median BSA was 1.48 (1.35–1.62) m^2^, with a range of 1.15–1.96 m^2^. A significant proportion (*n* = 25, 42.4%) met the low-BSA cutoff. The comorbidity burden was high, with a median CCI of 7.0 (5.5–8.0), and 51 patients (86.4%) had a CCI score of 5 or higher. Notably, 33 patients (55.9%) had chronic kidney disease (CKD) stage 5. This prevalence reflects the restricted use of LP in Thailand, with LP implantation largely reserved for patients with an absolute necessity, such as those with limited venous access.

### Low-BSA vs. Normal/High-BSA

Age and CCI were comparable between groups (Table 2); however, as expected, the low-BSA group had a significantly higher proportion of women than the normal/high-BSA group (84.0% vs. 23.5%, *P* < 0.001).

**Table 2:** Baseline characteristics and event comparisons between low-BSA and normal/high-BSA LP groups (*n* = 59)

Characteristic	Low BSA (*n* = 25)	Normal/High BSA (*n* = 34)	*P* value
Age, median (IQR), year	77.0 (68.0–82.0)	79.0 (70.0–83.0)	0.88
Women, No. (%)	21 (84.0)	8 (23.5)	< 0.001
BSA, median (IQR), m^2^	1.33 (1.28–1.37)	1.62 (1.53–1.71)	< 0.001
BMI, median (IQR), kg/m^2^	19.11 (16.87–20.54)	23.05 (31.62–25.22)	< 0.001
CCI, median (IQR)	7.0 (5.0–10.0)	7.0 (6.0–8.0)	0.42
CKD stage 5, No. (%)	16 (64.0)	13 (38.2)	0.21
Significant tricuspid valve disease, No. (%)	7 (28.0)	6 (17.6)	0.78

*Abbreviations*: *IQR* interquartile range, *CCI* Charlson Comorbidity Index, *BSA* body surface area, *BMI* body mass index, *CI* confidence interval

### Matched cohort (low-BSA LP vs. matched TVP)

The 25 low-BSA LP patients were matched 1:2 with 50 TVP patients. As intended, age, sex, and BSA were well-balanced (Table 3). However, the LP group exhibited a significantly higher comorbidity burden, including a higher median CCI (7.0 vs. 5.0, *P* = 0.002) and a significantly higher incidence of CKD stage 5 (64.0% vs. 4.0%, *P* = 0.006) compared to the matched TVP group.

**Table 3:** Baseline characteristics in the matched cohort analysis: low-BSA LP vs. 1:2 matched TVP

Characteristic	Low-BSA LP (*n* = 25)	TVP (*n* = 50)	*P* value^*^
Age, median (IQR), year	77.0 (68.0–82.0)	80.5 (73.2–85.0)	0.11
Women, No. (%)	21 (84.0%)	43 (86%)	0.37
BSA, median (IQR), m^2^	1.33 (1.28–1.37)	1.31 (1.25–1.36)	0.22
BMI, median (IQR), kg/m^2^	19.11 (16.87–20.54)	19.06 (16.72–20.79)	0.70
CCI, median (IQR)	7.0 (5.0–10.0)	5.0 (4.0–6.0)	0.002
AF, No. (%)	15 (60.0%)	29 (58.0%)	0.04
CVA, No. (%)	6 (24.0%)	5 (10.0%)	0.76
CKD stage 5, No. (%)	16 (64.0%)	2 (4.0%)	0.006
Diabetes, No. (%)	11 (44.0%)	16 (32.0%)	0.34
Hypertension, No. (%)	16 (64.0%)	33 (66.0%)	0.02
Significant tricuspid valve disease, No. (%)	7 (28.0%)	6 (12.0%)	0.78
Indication for PPM, No. (%)			0.001
Sinus node dysfunction	10 (40.0%)	33 (66.0%)	
High-grade AV block	15 (60.0%)	17 (34.0%)	

^*^ *P* value derived from conditional logistic regression stratified by matched-set identifier

*Abbreviations*: *AF* atrial fibrillation, *AV* atrioventricular, *BMI* body mass index, *BSA* body surface area, *CCI* Charlson Comorbidity Index, *CKD* chronic kidney disease, *CVA* cerebrovascular accident, *LP* leadless pacemaker, *PPM* permanent pacemaker, *TVP* transvenous pacemaker

### Procedural detail and outcomes

LP Implantation was successful in all but one patient (success rate, 98.3%), who developed cardiac perforation and required emergency cardiac surgery. All LP implantations were performed via femoral venous access: 53 (91.4%) via the right femoral vein and 5 (8.6%) via the left femoral vein. None of the patients underwent femoral vein imaging before or during the procedures because of local institutional practice and operator preference. The details of the models and the electrical parameters are listed in Table S1.

In the TVP group, the most common pacemaker system was the traditional dual-chamber pacemaker (*n* = 38, 76%), followed by conduction system pacing with dual-chamber pacemakers (*n* = 6, 12%), traditional single-chamber VVI (*n* = 4, 8%), and conduction system pacing with single-chamber VVI (*n* = 2, 4%). All implantation pockets were subcutaneous.

### Primary outcome: major complications

#### LP cohort analysis

Major complications occurred in 5 patients (8.5%), with 4 cases acute (occurring within 4 weeks) and 1 case late (occurring more than 4 weeks after the procedure). There were no procedure-related deaths. Details of all complications are provided in Table 4.

**Table 4:** Details of the major complications in all cohorts

Cohorts	Event Type (No.)	Description	Outcome
Normal/High-BSA LP	Pericardial effusion (1)	Pericardial effusion with cardiac tamponade physiology; required pericardiocentesis.	Recovered without sequelae.
Normal/High-BSA LP	System revision (1)	Premature battery depletion (< 3 years) due to high pacing threshold (> 2 V at 0.24ms); required new device implantation.	Successful replacement.
Low-BSA LP	Cardiac perforation (1)	Right ventricular perforation required emergent surgical repair.	Recovered with neurological deficit.
Low-BSA LP	System revision (1)	Intermittent loss of capture at maximum output due to high pacing threshold at day 5; required device repositioning.	Successful repositioning.
Low-BSA LP	Pulmonary embolism (1)	Acute dyspnea without significant hemodynamic compromise. Treated with anticoagulation.	Recovered without sequelae.
Matched TVP	Pneumothorax (3)	Three separate cases; all conventional DDD; all required chest tube drainage.	All resolved.
Matched TVP	Lead dislodgement (3)	Three separate cases required revisions: 1. One ventricular lead of a CSP VVI 2. One ventricular lead of a conventional VVI 3. One atrial lead of a conventional DDD	All successful revised.
Matched TVP	Embolic stroke (1)	Massive stroke occurring while anticoagulation was withheld (conventional DDD).	Death (day 5)

*Abbreviations*: *BSA* body surface area, *CI* confidence interval, *CSP* conduction system pacing, *LP* leadless pacemaker, *TVP* transvenous pacemaker

In the low-BSA group (*n* = 25), three patients (12%) experienced complications: one had cardiac perforation requiring emergency surgical repair, one required system revision due to elevated pacing thresholds, and one developed a pulmonary embolism. In the normal/high-BSA group (*n* = 34), two complications (5.9%) occurred: one pericardial effusion requiring drainage and one premature battery depletion due to a high threshold, necessitating implantation of a new device. Despite the numerical difference in raw events, there were no statistically significant differences between the low-BSA and normal/high-BSA groups in the 3.8-year cumulative incidence of major complications (8.0% vs. 8.8%, *P* = 0.39).

#### Matched cohort analysis

Major complications occurred in 3 (12%) patients in the low-BSA LP group and 7 (14%) in the TVP group. There was no significant difference in the 3.8-year cumulative incidence of major complications between the two cohorts (8.0% vs. 14.0%, *P* = 0.91). Complications in the TVP group included pneumothorax (*n* = 3), lead revision (*n* = 3), and an embolic stroke (*n* = 1). Of these TVP events, two occurred in patients with single-chamber systems and five in those with dual-chamber systems (Table 4).

**Fig. 2:**
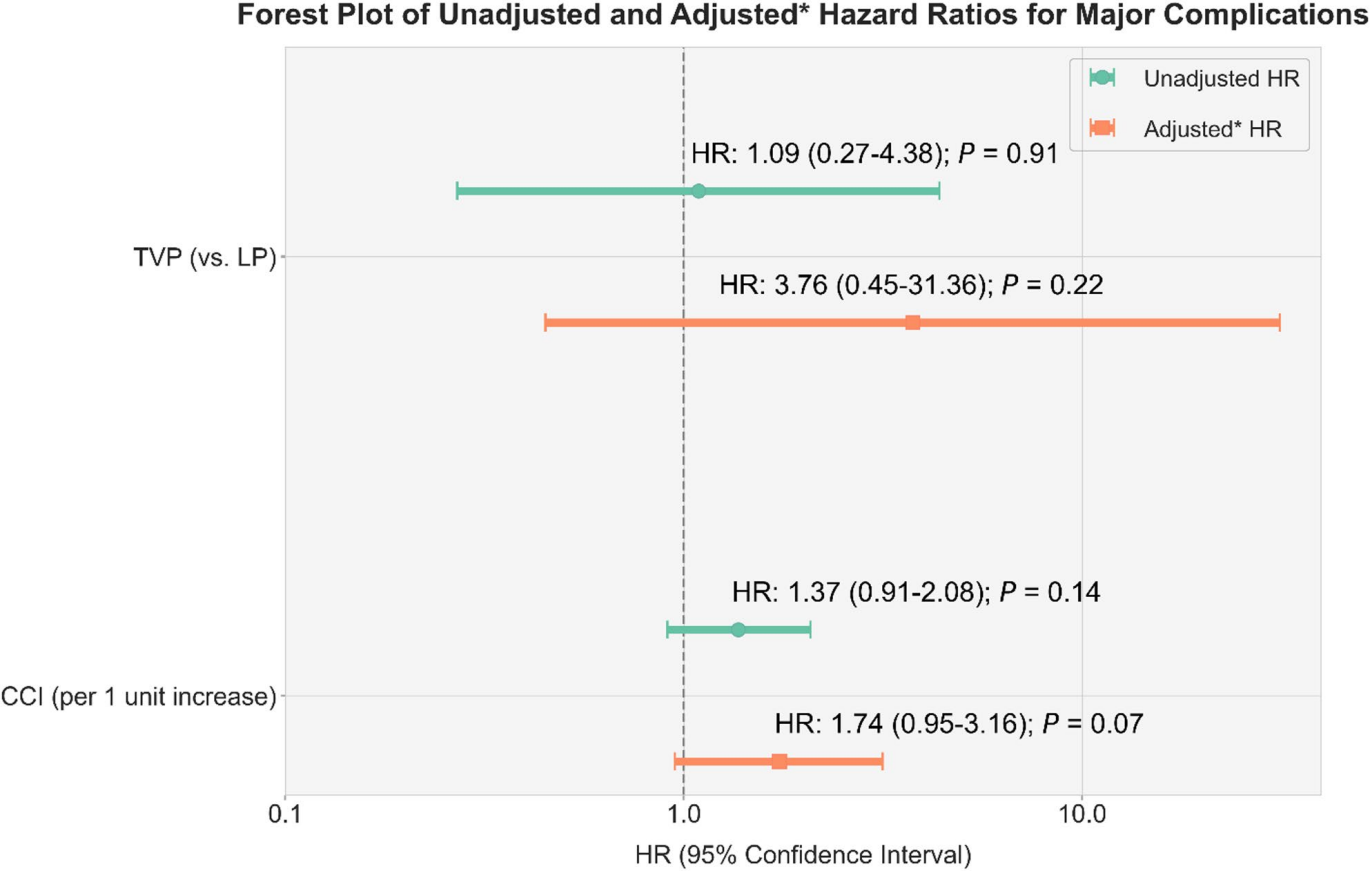
Forest plot of unadjusted and adjusted hazard ratios for major complications of the matched cohort. The analysis employed the Cox proportional hazards method, stratified by the matched-pair identifier to account for the study design, and was analyzed in a cause-specific manner to account for the competing risks of death and major complications. The adjusted model was controlled for the Charlson comorbidity index (CCI). The plot displays hazard ratios and their corresponding 95% confidence intervals for two variables: pacemaker type (transvenous pacemaker vs. leadless pacemaker) and CCI, modeled as a continuous variable per unit increase. * Adjusted model controlled for CCI. Abbreviations: CCI, Charlson Comorbidity Index; HR, hazard ratio; LP, leadless pacemaker; TVP, transvenous pacemaker

The unadjusted stratified cause-specific Cox model revealed no significant difference in the hazard of complications between the TVP and LP groups (HR, 1.09; 95% CI, 0.27–4.38; *P* = 0.91). After adjusting for CCI to account for baseline imbalance, the point estimate for the HR for the TVP group relative to the low-BSA LP group increased; nonetheless, the association remained nonsignificant with a wide confidence interval (aHR, 3.76; 95% CI, 0.45–31.36; *P* = 0.22). In the same model, a one-point increase in CCI was associated with a borderline significant increase in the hazard of complications (aHR, 1.74; 95% CI, 0.95–3.16; *P* = 0.07). These findings are illustrated in the forest plot shown in Fig. 2.

### Secondary outcome: all-cause mortality

During a median follow-up of 1.4 (0.2–3.3) years, 13 patients (22.0%) in the whole LP cohort died. There were no statistically significant differences between the low-BSA and normal/high-BSA groups in the 3.8-year cumulative incidence of all-cause mortality (24.5% vs. 48.4%, *P* = 0.70).

In the matched analysis, six (24.0%) and six (12.0%) deaths occurred in the low-BSA LP and TVP groups, respectively. The majority of deaths in both groups were non-cardiovascular (Table S2). There was no statistically significant difference in the unadjusted (HR, 0.24; 95% CI, 0.05–1.25; *P* = 0.09) or adjusted (aHR, 0.12; 95% CI, 0.01–1.67; *P* = 0.11) hazard of death between the TVP and low-BSA groups.

## Discussion

In this exploratory analysis of patients with high comorbidities who underwent LP implantation, low BSA was not associated with an increased risk of major complications or all-cause mortality. This finding was consistent with both internal (low-BSA LP vs. normal/high-BSA LP) and external comparisons against a pair-matched TVP cohort (1:2 matched for age, sex, and BSA).

This study aimed to directly address the safety concerns surrounding LP implantation in patients with a small body habitus, a concern particularly relevant to the Asian context. Quantitative studies measuring the diameter of the common femoral vein have demonstrated that individuals of Asian ethnicity have the smallest average diameters, even after adjusting for age and sex [17]. Furthermore, because LP is not fully reimbursable in Thailand and costs significantly more compared to TVP, its use is judiciously reserved for patients with clinical indications to avoid a lead or pocket. Consequently, our cohort at this super-tertiary center represents a highly selected group characterized by severe illness (median CCI of 7, 56% having CKD stage 5), advanced age (median age, 79 years), and frailty (median BSA, 1.48 m²; with the lowest at 1.15 m²). Overall, 45 patients (76.3%) exhibited specific LP indications, most notably limited venous access (*n* = 35, 59.3%) and a history of TVP infection (*n* = 8, 13.6%).

We selected BSA as the primary anthropometric metric because it is directly correlated with the size of the cardiac chambers and major blood vessels [10–12]. While other metrics, such as weight, height, or BMI, may be considered, studies have demonstrated that BSA is a superior proxy, showing a closer relationship with the diameter of the common femoral vein and the size of the left ventricle [10, 11]. The formula of Yu et al.^13^ was used to calculate BSA. It was developed using a 3D scanner to measure BSA in Chinese adults and is widely regarded as the most accurate formula [18]. The cut-point value of 1.46 m² for low BSA was chosen from the same study of Chinese participants, who are expected to closely resemble our population [14]. This value is equal to 1.51 m^2^ and 1.53 m^2^ using the Dubois and Mosteller formulas, respectively [13, 18]. 

The major complication rates in our LP patients—crude and cumulative incidences of 5.8% and 8.0%, respectively—were substantially higher than the approximately 4% incidence reported in the comprehensive, less-selected Micra transcatheter study [2]. Nevertheless, our complication rates align with previous data analyzing LP outcomes in high-risk subgroups. In a real-world study,^4^ these high-risk subgroups demonstrated acute complication rates ranging from 8.6% to 14.5%, depending on the type of comorbidity. When focusing specifically on patients with small body habitus, data are limited, as large registries primarily enroll patients with higher body mass (e.g., a mean BMI of 27.6 kg/m^2^ in the Micra Transcatheter Pacing Study [1]). A more direct comparison was reported in regional Asian studies. In a low-BMI cohort of Micra LPs from Hong Kong (mean BMI, 20.93 kg/m^2^; mean BSA [Mosteller], 1.51 m^2^), the major complication rate was 5.6% (4 of 71 patients), with one death [7]. In another cohort from Hong Kong using the Aveir LPs [19] (mean BMI, 22.5 kg/m^2^; mean BSA [Mosteller], 1.60 m^2^), the acute complication was 1.6% (2 of 123 patients). Our low-BSA group had a comparable body habitus. The median BMI and BSA were 19.11 kg/m² and 1.33 m² (equivalent to 1.40 m² by Mosteller), respectively. However, our cohort was far more severely ill, with 64% of patients having CKD stage 5, compared to less than 5% in the aforementioned studies. Despite this extreme comorbidity, the major complication rate in the low BSA LP group was 12% (3 of 25 patients), which is still within the range of high-risk cohorts [4] and, importantly, occurred without any deaths. Furthermore, we demonstrated the technical feasibility of our smallest patient, who weighed 35 kg (BMI 15.6 kg/m^2^; BSA 1.15 m^2^) and underwent the procedure without complications, consistent with the findings of a published case report [20] of successful LP implantation in a patient with a small body habitus.

The low number of LP implantations at our center (59 cases from 2016 to 2025) relative to TVP implantations (1,131 cases during the same period) reflects a restrictive reimbursement policy. To maximize the statistical power while preserving the LP cohort, we employed a pair-matched analysis controlling for age, sex, and BSA to reduce confounders without excluding patients with LP. Notably, compared with those with TVP, patients with LP remained significantly sicker at baseline despite demographic and anthropometric matching, with a higher CCI and CKD stage 5 prevalence. In the comparative analysis of major complications, the crude and cumulative incidences of major complications were numerically lower in the low-BSA LP group than in the matched TVP group. The unadjusted HR of major complications comparing TVP over low-BSA LP was nonsignificant (HR, 1.09; 95% CI, 0.27–4.38; *P* = 0.91). Nonetheless, upon adjusting for CCI, the risk estimate increased sharply (aHR, 3.76; 95% CI, 0.45–31.36; *P* = 0.22). Although this finding remained nonsignificant, likely due to sparse outcome events, which widened the CIs, the adjusted model provided strong contextual insight: CCI itself was a borderline significant major complication predictor (aHR, 1.74; 95% CI, 0.95–3.16; *P* = 0.07). Thus, patient comorbidities rather than the choice of pacemaker system or small body size may be critical determinants of procedural risk in this highly selected population. These results align with those of the Micra Coverage with Evidence Development study, a larger and non-selected study using US Medicare claims data, which found that the incidence of acute complications did not differ between LP and TVP [21]. Importantly, LP demonstrated long-term safety advantages, with significantly lower rates of infection and reintervention at 3 years [3]. 

Because the LP system also features an atrial-sensing model, we included both single- and dual-chamber implantations in the TVP cohort to represent real-world practice. Although it may be argued that dual-chamber systems inherently carry an increased complication risk due to the presence of an additional lead, modern registry data [22, 23] suggest that the overall rate of major complications is relatively similar between the two systems. Specifically, the rates of pericardial complications and pneumothorax were comparable; however, the single-chamber system was associated with fewer pocket- or lead-related issues but a higher need for system upgrades. Furthermore, limiting the TVP cohort to single-chamber systems would have compromised the integrity of the pair-matching analysis due to their low volume (approximately one-tenth of the dual-chamber implantations at our center). The complications observed in our TVP group were distributed fairly across both systems (two in the single-chamber and five in the dual-chamber), supporting the decision to pool the implantations.

The primary strengths of our study include its unique focus on low BSA, the use of a strict pair-matched analysis (controlling for age, sex, and BSA), and the granular data obtained from a high-comorbidity patient population that is typically underrepresented in large registries. These findings suggest that underlying comorbidities, rather than small body size or pacemaker type alone, may be the primary drivers of procedural risk in this challenging group.

Our study has several limitations. First, this is a single-center retrospective analysis with a small sample size (*n* = 59 for all LPs and *n* = 25 in the low-BSA LPs), resulting in wide confidence intervals and limited statistical power. Second, the comparison group was matched for age, sex, and BSA; however, significant clinical imbalances persisted. Notably, the low-BSA LP group had a higher CCI score and a higher incidence of CKD stage 5 than the matched TVP group, introducing residual confounding. Finally, pre-procedural venous imaging was not performed according to local institutional practice and operator preference, precluding a direct anatomical correlation between vein size and vascular complications. Consequently, these findings should be viewed as exploratory and hypothesis-generating rather than definitive.

## Conclusion

In this exploratory analysis of high-risk, low-BSA patients, LP implantation, when performed by experienced operators, showed no increased risk of major complications compared to a matched TVP cohort. However, the small sample size and significant baseline comorbidities in the low-BSA LP patients caused pronounced selection bias, precluding definitive conclusions regarding safety equivalence. These findings support the use of LP in small patients but highlight the need for caution and larger prospective studies.

## Supplementary Information

Supplementary Material 1.

## Data Availability

The datasets generated and analyzed during this study are not publicly available due to institutional privacy policies, but they can be obtained from the corresponding author upon reasonable request.

## Abbreviations

aHR: adjusted hazard ratio
AF: atrial fibrillation
AJ: Aalen–Johansen
AV: atrioventricular
BMI: body mass index
BSA: body surface area
CCI: Charlson Comorbidity Index
CI: confidence interval
CKD: chronic kidney disease
CVA: cerebrovascular accident
HR: hazard ratio
IQR: interquartile range
LP: leadless pacemaker
PPM: permanent pacemaker
TVP: transvenous pacemaker
