# Supplementary material for: Safety of leadless versus transvenous pacemakers in patients with low body surface area: a matched-pair analysis
Source: BMC Cardiovasc Disord. 2026 Jan 16;26:138. doi: 10.1186/s12872-026-05526-0 (PMC12892651; doi:10.1186/s12872-026-05526-0)
Supplement: Supplementary file 1 — Supplementary Material 1. [file 12872_2026_5526_MOESM1_ESM.docx]

**Table S1.** Models and electrical parameters of all patients with leadless pacemakers (n = 59)

| **Characteristic** | **Data Value** |
| --- | --- |
| Models of leadless pacemakers, No. (%) |  |
| Micra MC1VR01 | 45 (76.3) |
| Micra MC1AVR1 | 10 (16.9) |
| Micra MC2VR01 | 3 (5.1) |
| Micra MC2AVR1 | 1 (1.7) |
| Electrical Parameters at Implantation |  |
| Threshold, mean (SD), Voltage at 0.24 ms | 0.6 (0.4) |
| Impedance, mean (SD), Ω | 723.6 (160.2) |
| Electrical Parameters at 4 weeks |  |
| Threshold, mean (SD), Voltage at 0.24 ms | 0.6 (0.4) |
| Impedance, mean (SD), Ω | 609.4 (137.2) |
| Ventricular pacing, median (IQR), % | 30.0 (16.1–98.5) |
| SD, standard deviation; IQR, interquartile range. |  |

**Table S2.** Causes of death in stratified by study cohorts.

| **Causes of Death** | **All LPs (n = 59)** | **Low-BSA LPs (n = 25)** | **Matched TVPs (n = 50)** |
| --- | --- | --- | --- |
| **Cardiovascular, No. (%)** |  |  |  |
| Heart failure | 1 (1.7) | 0 (0.0) | 1 (2.0) |
| Arrhythmia | 0 (0.0) | 0 (0.0) | 1 (2.0) |
| Cerebrovascular accident | 0 (0.0) | 0 (0.0) | 1 (2.0) |
| **Non-cardiovascular, No. (%)** |  |  |  |
| Infection/Sepsis | 3 (5.1) | 2 (8.0) | 1 (2.0) |
| Malignancy | 1 (1.7) | 1 (4.0) | 0 (0.0) |
| Pulmonary | 3 (5.1) | 2 (8.0) | 0 (0.0) |
| Gastrointestinal | 2 (3.4) | 0 (0.0) | 0 (0.0) |
| Metabolic | 1 (1.7) | 0 (0.0) | 0 (0.0) |
| **Undetermined, No. (%)** | 2 (3.4) | 1 (4.0) | 2 (4.0) |
| **Total Deaths, No. (%)** | **13 (22.0)** | **6 (24.0)** | **6 (12.0)** |

Abbreviations: BSA, body surface area; LP, leadless pacemaker; TVP, transvenous pacemaker
